# Supplementary material for: Dimensionality-reduction techniques for complex mass spectrometric datasets: application to laboratory atmospheric organic oxidation experiments
Source: Atmos Chem Phys. Author manuscript; Available in PMC 2021 Mar 26. (PMC7995644; doi:10.5194/acp-20-1021-2020)
Supplement: Supplement [file NIHMS1662563-supplement-Supplement.pdf]

Supplement of Atmos. Chem. Phys., 20, 1021–1041, 2020
https://doi.org/10.5194/acp-20-1021-2020-supplement
© Author(s) 2020. This work is distributed under
the Creative Commons Attribution 4.0 License.

Atmospheric Chemistry and Physics
Open Access

Supplement of

Dimensionality-reduction techniques for complex mass spectrometric datasets: application to laboratory atmospheric organic oxidation experiments

Abigail R. Koss et al.

Correspondence to: Abigail R. Koss (abigail.r.koss@gmail.com)

The copyright of individual parts of the supplement might differ from the CC BY 4.0 License.

Figure S1

Ions measured by Vocus-2R-PTR during chamber experiment.

A. Kendrick mass defect plot of unambiguously identified ions (Vocus-2R-PTR instrument).

B. Kendrick mass defect plot of all ions.

Markers are sized and colored by peak area. In subplot A, a line has been drawn through large, unambiguously identified peaks $C_9H_{13}O_n^+$ with *n* between 1 and 4. In subplot B, the series has been extended to include *n*>4. The identities of other peaks with *m/z* >200 were suggested in a similar way, by identifying trends in ion formulas with m/z<200 and extending the series to larger *m/z*.

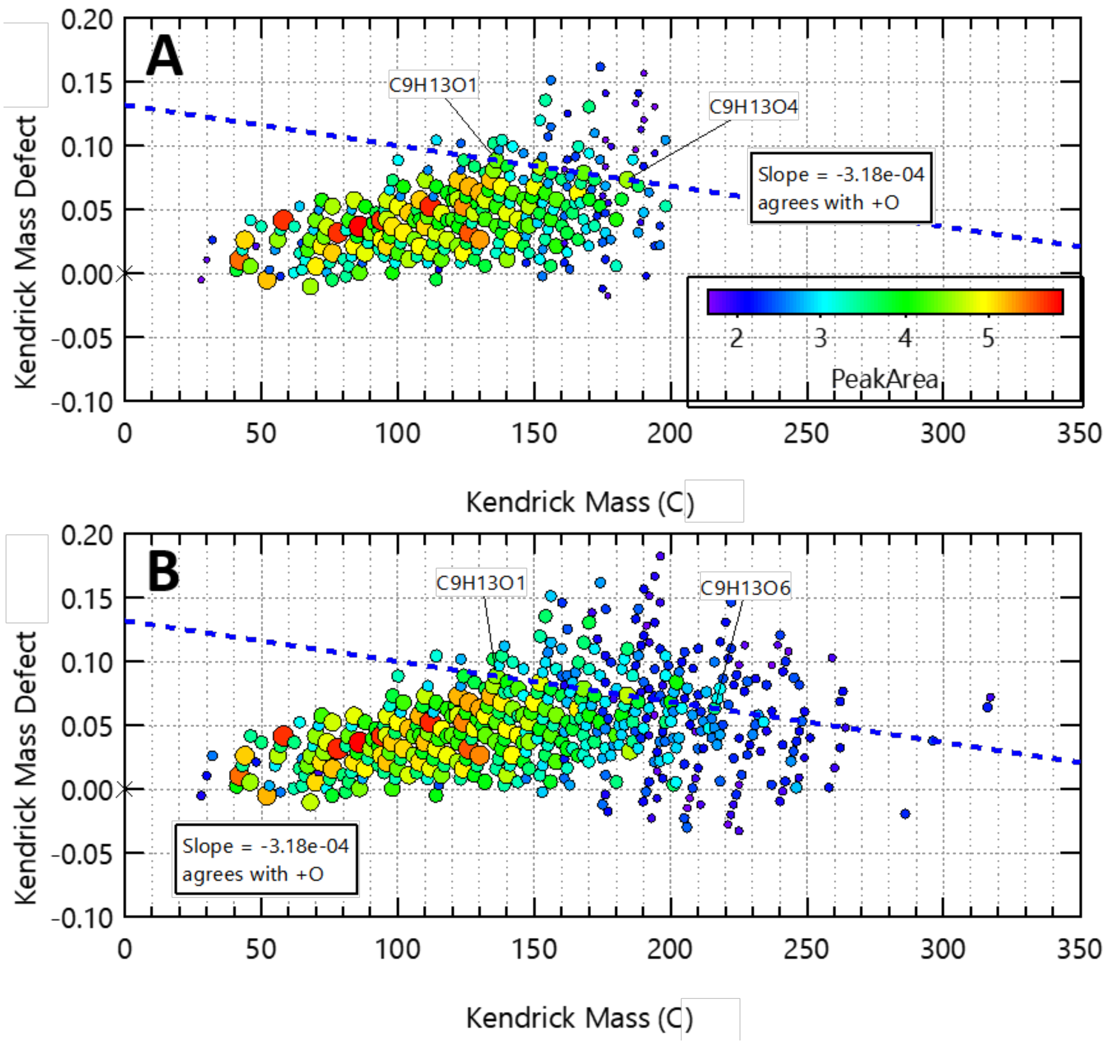

Figure S2

Identification of chemically relevant ions within a mass spectrum (here, from the PTR3 H_3O^+ instrument during a chamber experiment) using hierarchical clustering analysis. A. Average mass spectrum showing chemically relevant ions in red and non-relevant ions in gray. B. Hierarchical cluster of ions. Relevant ions and the clusters they belong to are highlighted in red. Boxes are drawn around two example clusters, “C,” which includes non-relevant ions, and “D,” which includes relevant ions. C. Time series of all ions belonging to cluster “C.” The average is drawn in black. D. Time series of all ions belonging to cluster “D.” The average is drawn in black. The oscillating pattern is due to ion source stabilization after reagent-ion switching and was not included in final data. 1330 ions were detected and quantified. Of these, 251 have time-dependencies consistent with products of the oxidation of 1,2,4-trimethylbenzene. Ten to twenty clusters were visually inspected to identify which ions should be excluded from further analysis.

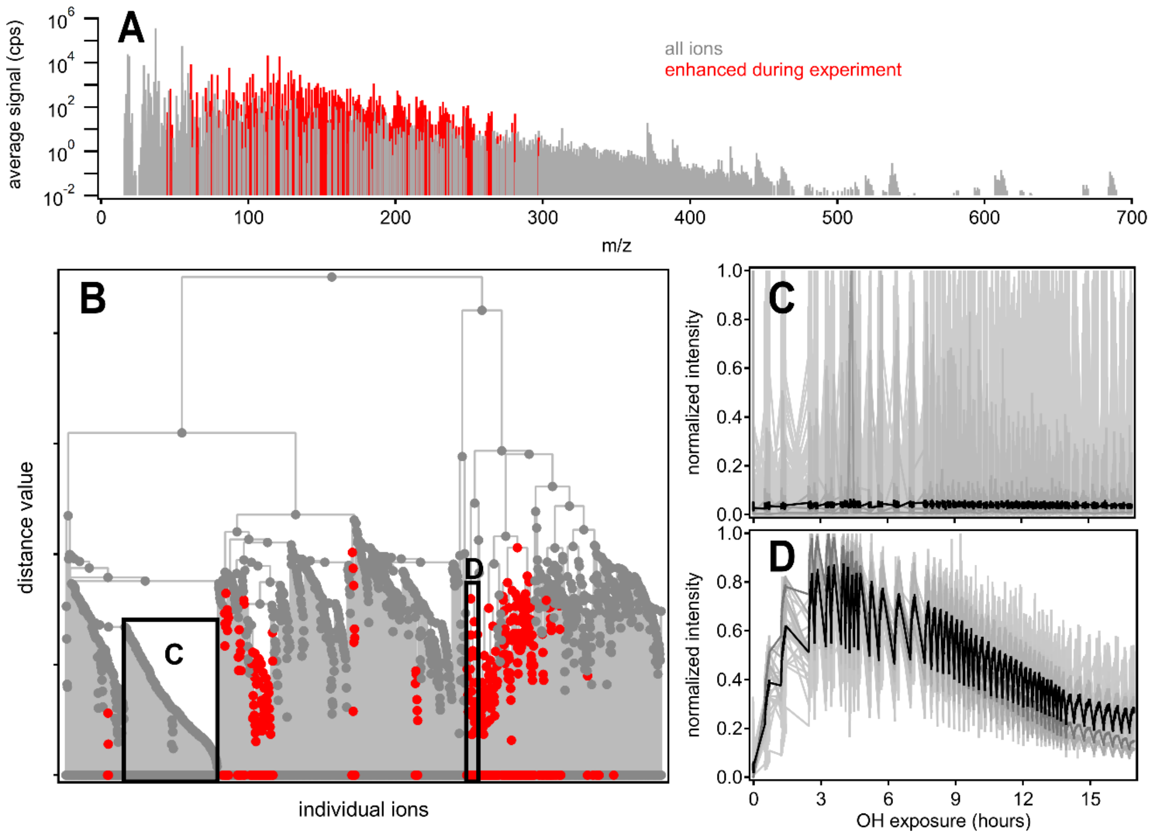

Figure S3

Signal-to-noise ratios for the synthetic data system (left) and chamber data (right). For PMF, species with SNR<2 are downweighted by a factor of 2, and species with SNR<0.2 are downweighted by a factor of 10.

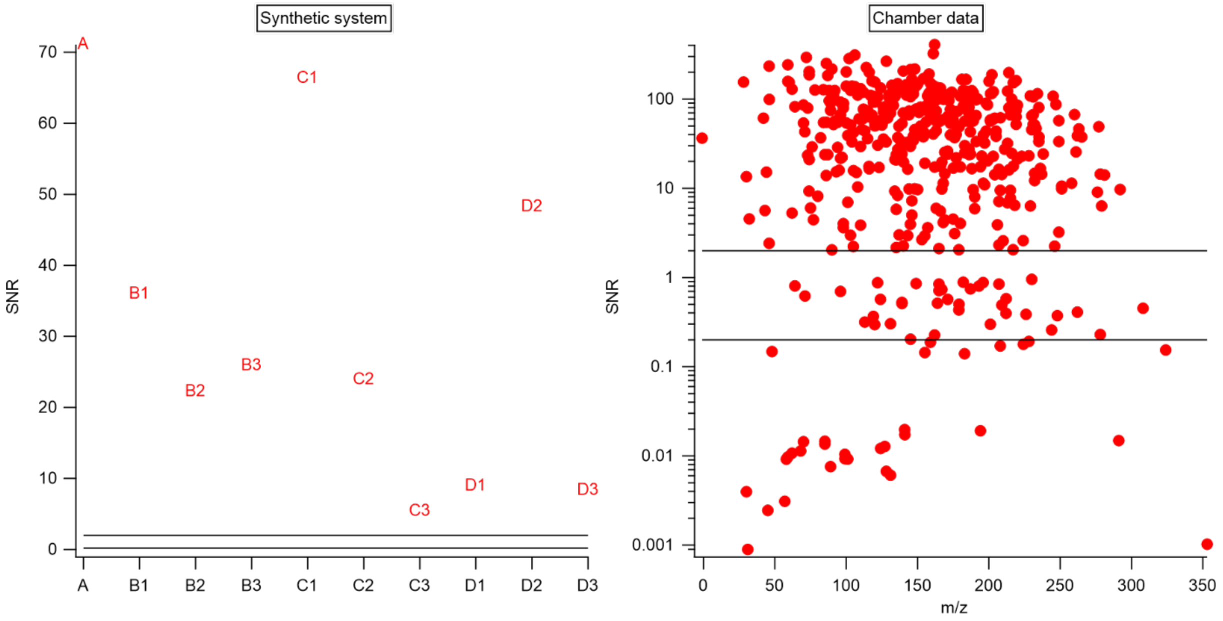

Figure S4

Relationship between standard deviation and signal for all chamber measurements.

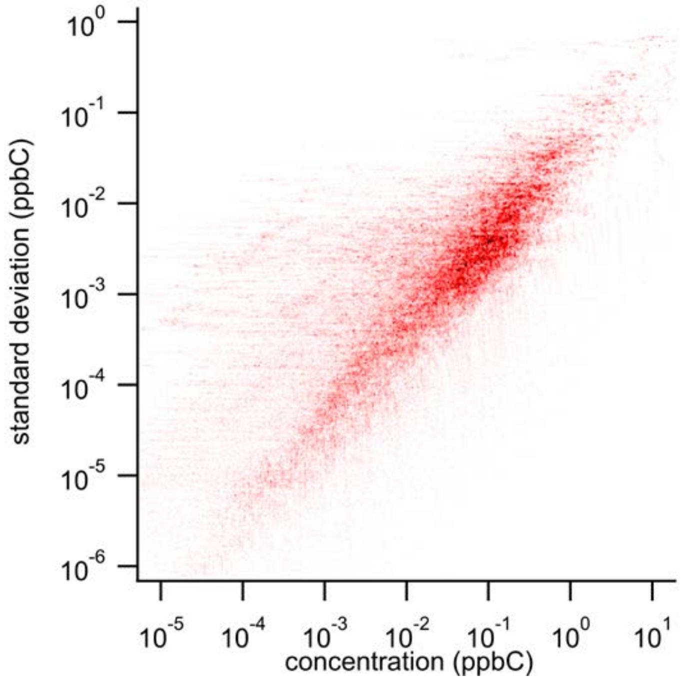

Figure S5

Time series and factor profiles of PMF analysis of synthetic data

Synthetic system: solution as a function of number of factors. Factor profiles

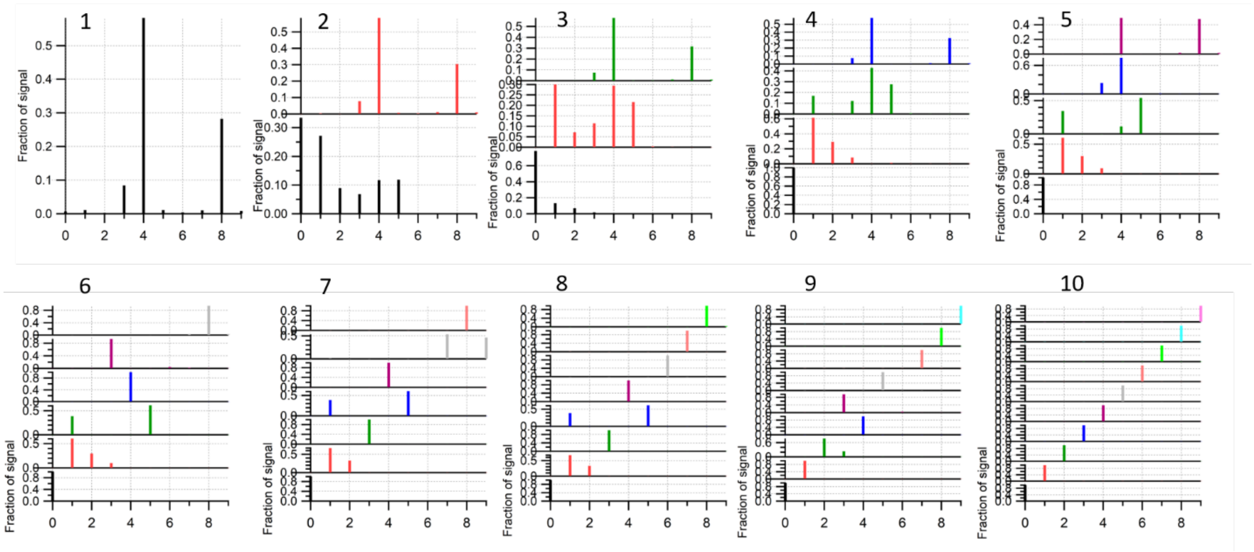

Synthetic system: solution as a function of number of factors. Time series

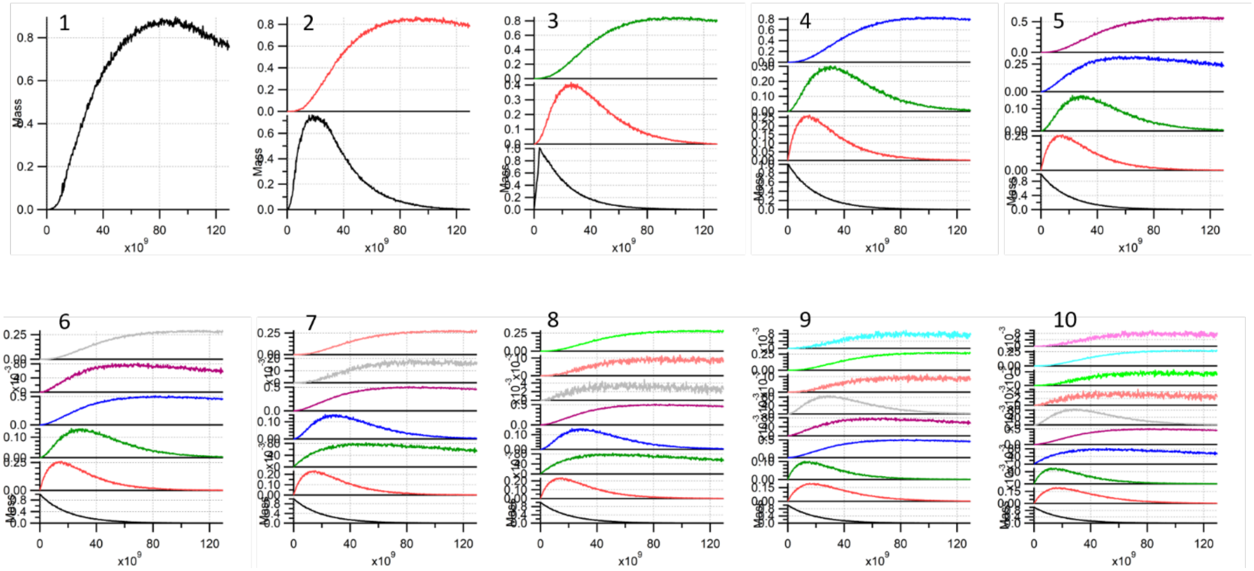

Figure S6

PMF results for chamber data. Three- to eight-factor solutions are shown. No solution was found for two factors.

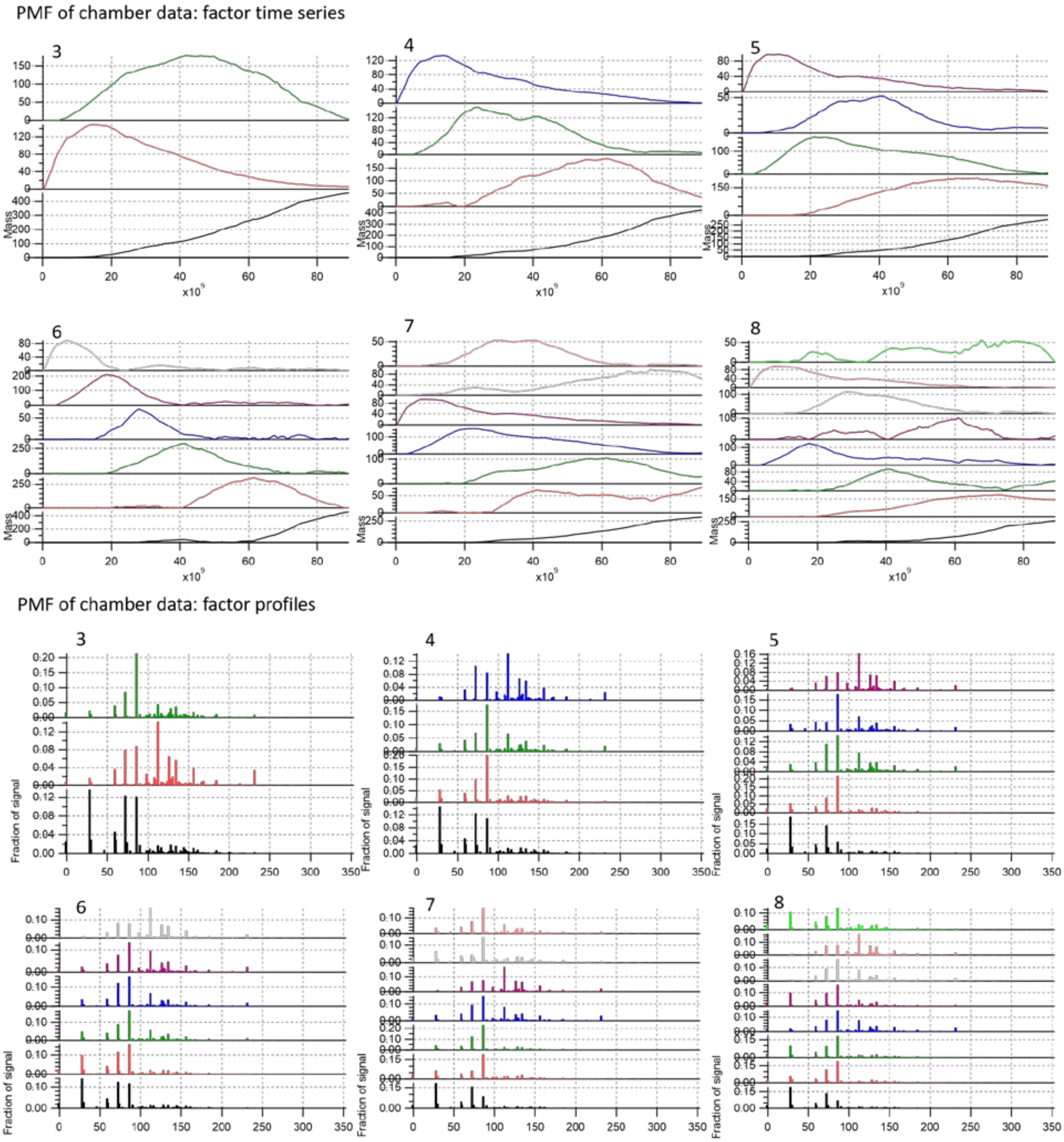

Figure S7

Time series of all clusters and individual species from HCA analysis of chamber data. Individual species are shown as thin lines. Cluster averages are shown as thick lines, and the individual species contributing to that cluster are included as thin gray lines. In each plot, the y-axis is normalized intensity and the x-axis is OH exposure.

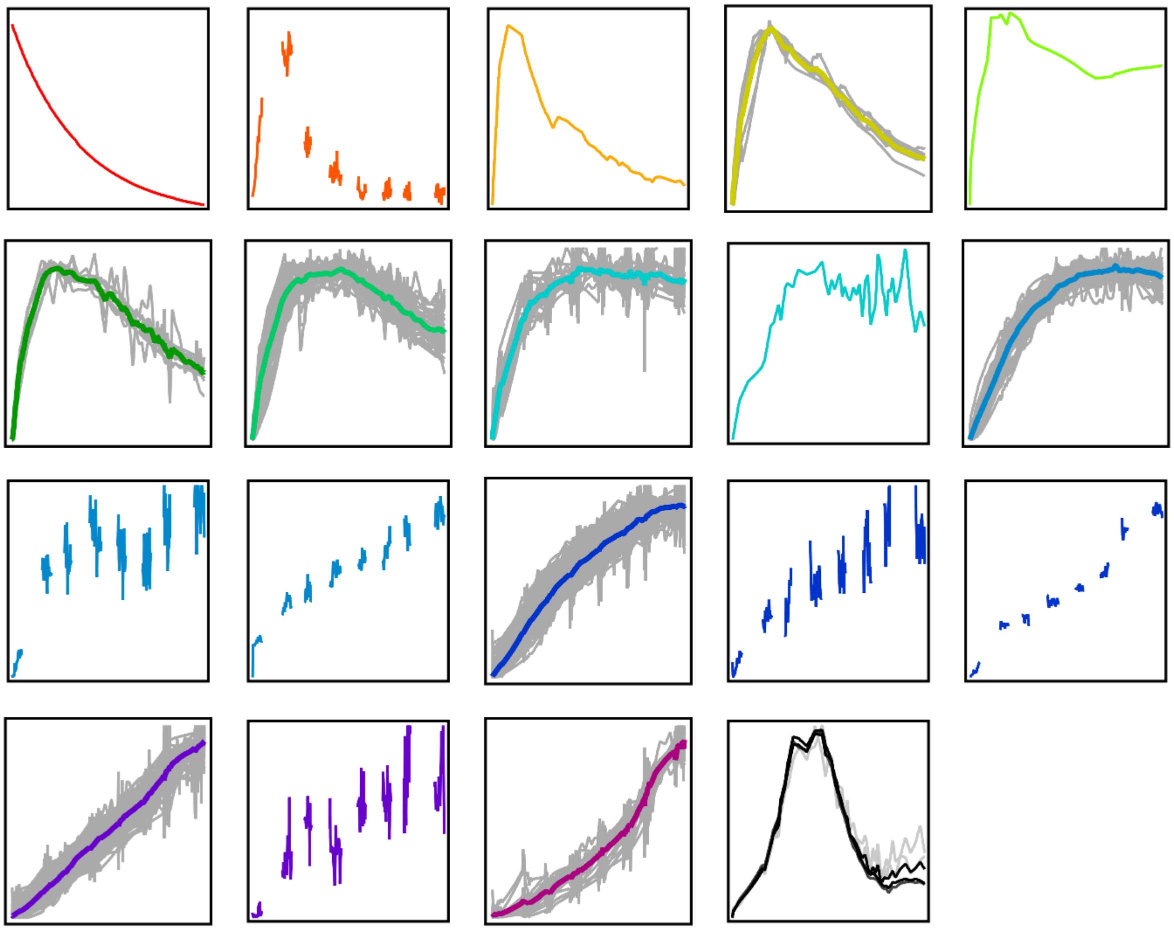

Figure S8
Clustering results of chamber data at different relative distances.

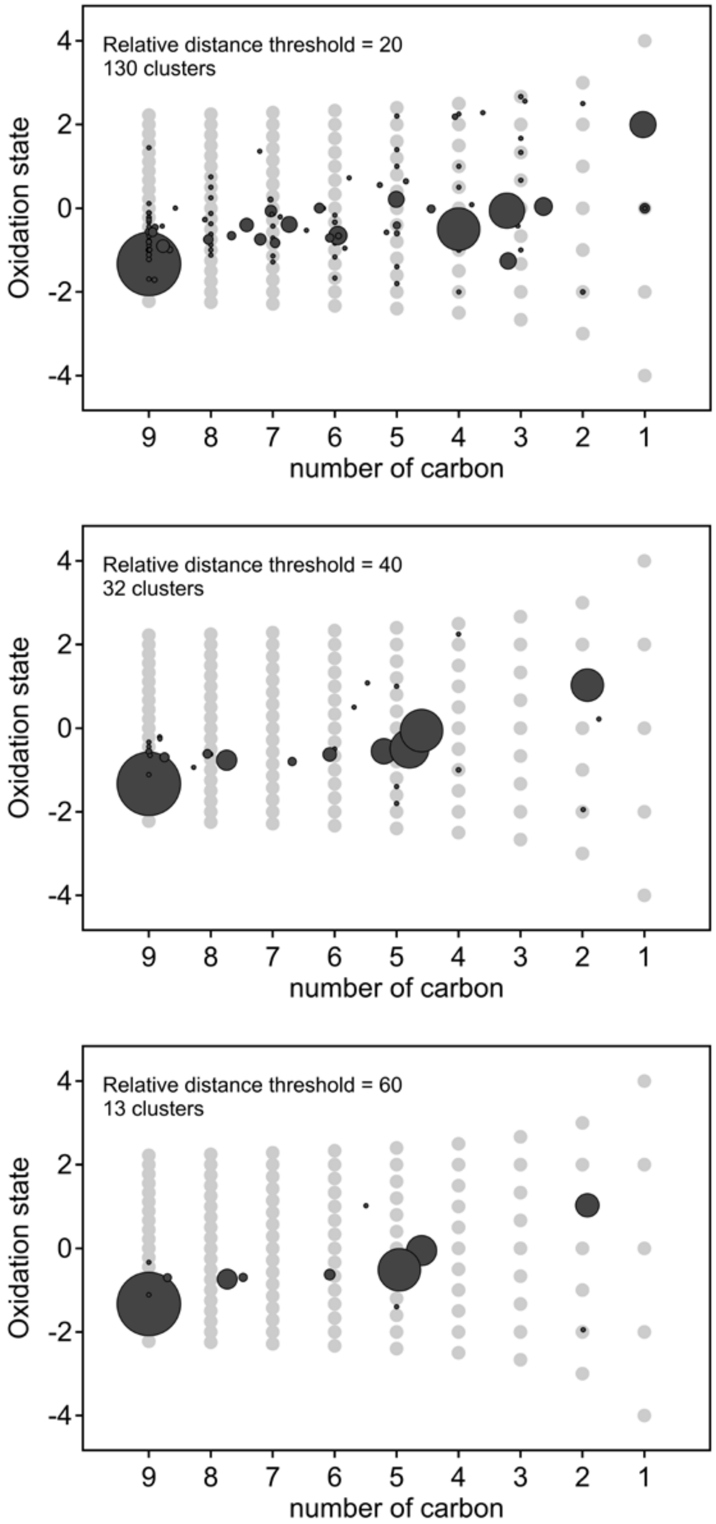

Figure S9

Standard deviation of fit parameter for *m* and *k.* Chamber data is shown.

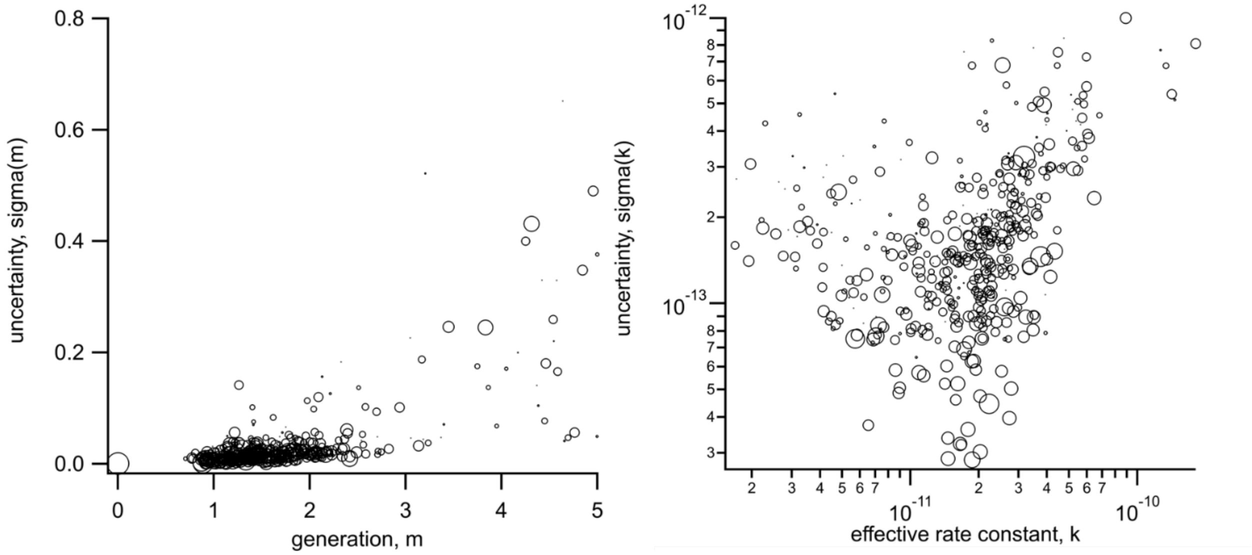

Figure S10

Parameterized generation for non-linear systems, using synthetic data.

The reaction pathway for two different synthetic systems is shown at the top. The rate constants are in units of 10^{-11} cm^3 $molecule^{-3}$ s^{-1}.

A. Time series of reactant species in synthetic system 1. B. Parameterized generation numbers for synthetic system 1. C. Time series of reactant species in synthetic system 2. D. Parameterized generation numbers for synthetic system 2.

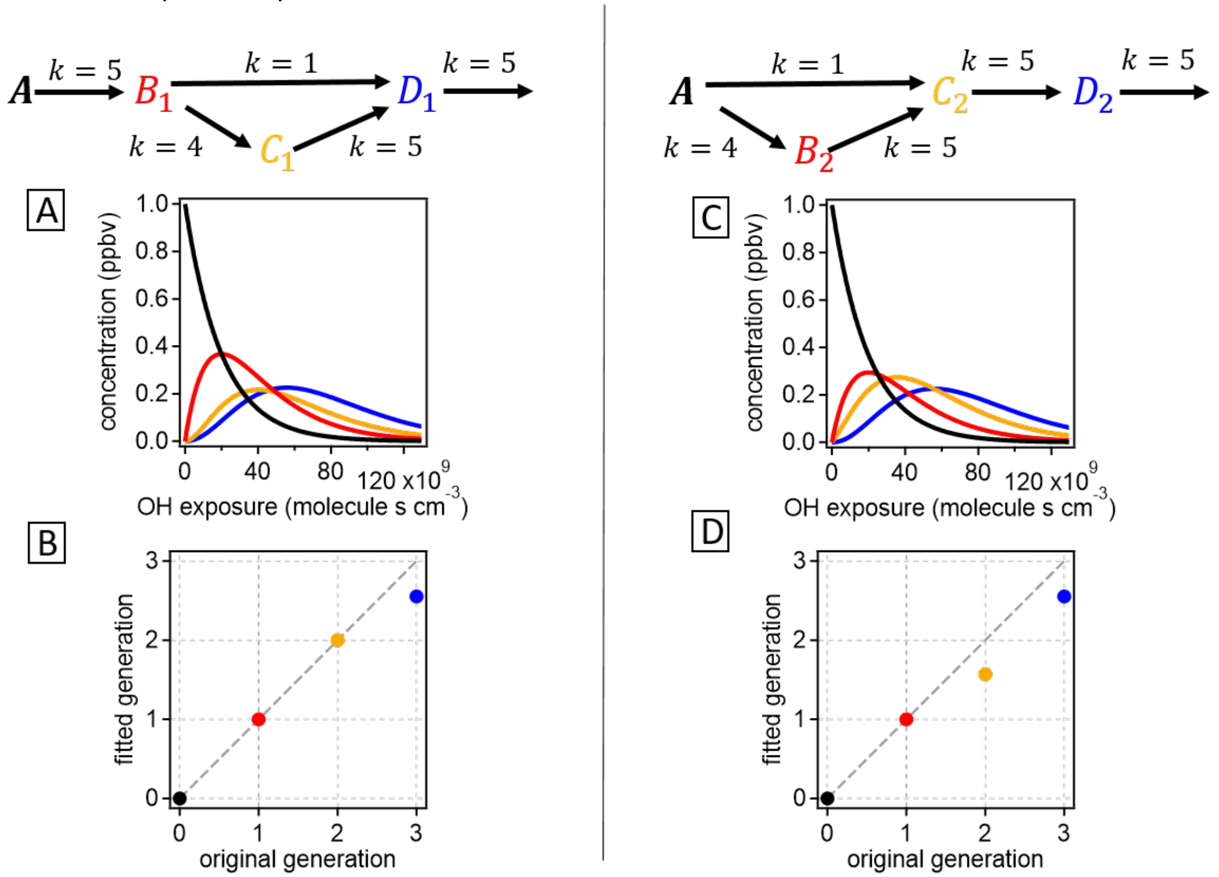

S1 Best methods for determining generation number

The best fit parameterization of *m* can be improved with two methods: one, by fitting to early data; and two, by reducing noise.
The generation number *m* is determined from the curvature of the initial growth of the product species. Based on the method of fitting, the curve fit algorithm can return an incorrect value of *m*. For example, the following two species were fit using least squares, which is the default method in many software packages.

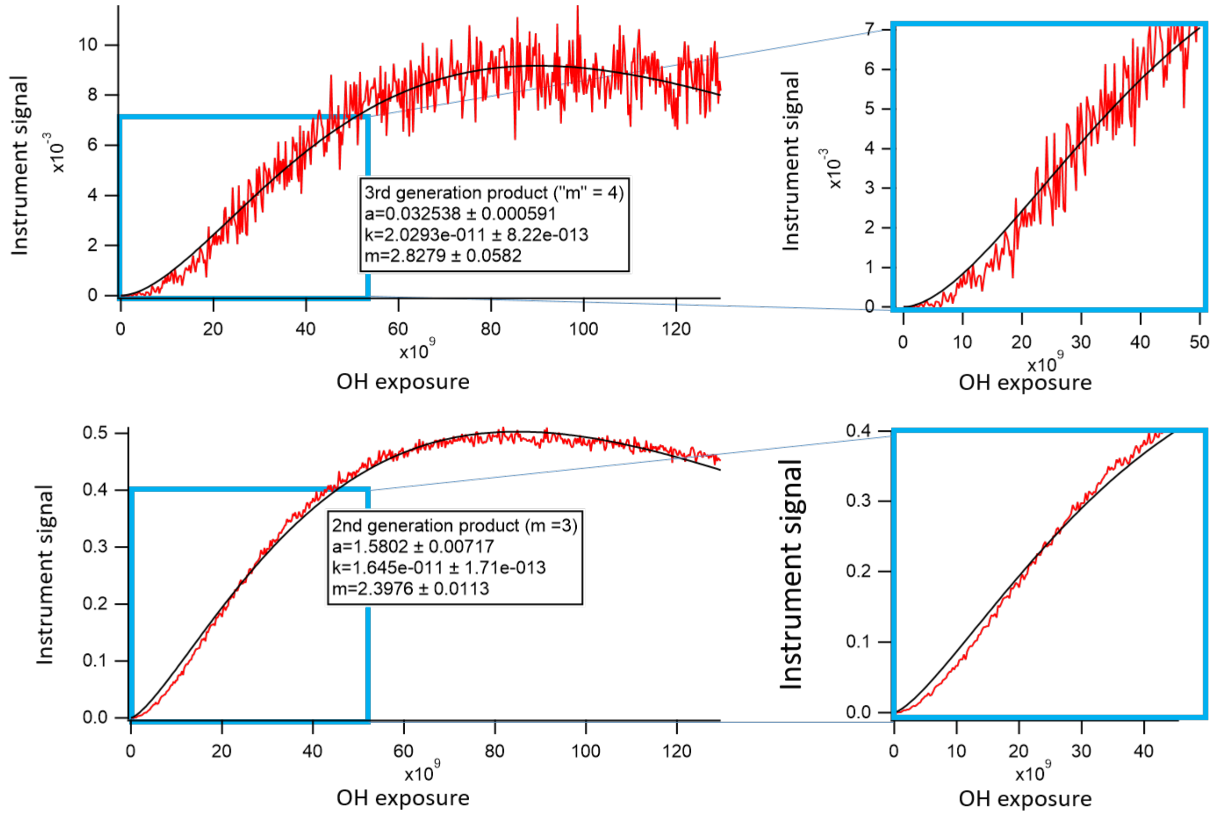

Figure S1.1

The left two panels of Figure S1.1 show the full time series of synthetic data, and the right two panels expand the boxed inset. This figure shows that the fit is poorer for early time points. Later data are fit better, because they have higher values and are therefore weighted more heavily in least-squares fitting. The result is an artificially low returned value of *m*. This issue can be solved by fitting to early data only. The optimal number of points to fit differs based on the *k* and *m* of the species in question. If two few points are fit, then no trend is discernable; if too many points are fit, then *m* is underpredicted.

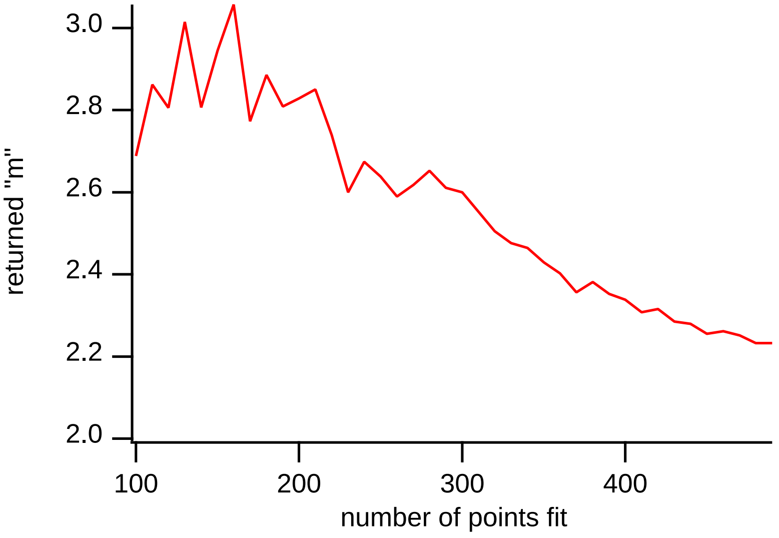

Figure S1.2

Figure S1.2 shows the returned value of *m* as a function of number of data points fit, using species "C3" from the synthetic system as an example. Based on the typical noise level of our data, we chose to exclude fits with fewer than 100 data points. The largest returned value of *m* is the most accurate.

The fit can be further improved by reducing noise. Mass spectrometers typically exhibit a Poisson noise distribution, where values are normally distributed about the actual signal. This noise should cancel out in the integration of a measurement, resulting in a smoother curve. The integral of Eq. 2 is:

$\int [X]dt = \frac{a}{k}\left(1 - \frac{\Gamma(m,kt)}{\Gamma(m)}\right)$ (Eq. S1)

where $\Gamma(m, kt)$ and $\Gamma(m)$ are the partial and complete gamma functions, respectively. Eq. S1 can be fit to integrated data, using for time *t* the OH exposure *OHΔt*. The returned values of *m* as a function of points fit, using integrated data, is shown in Figure S1.3. This returns a more accurate value of *m* using fewer data points.

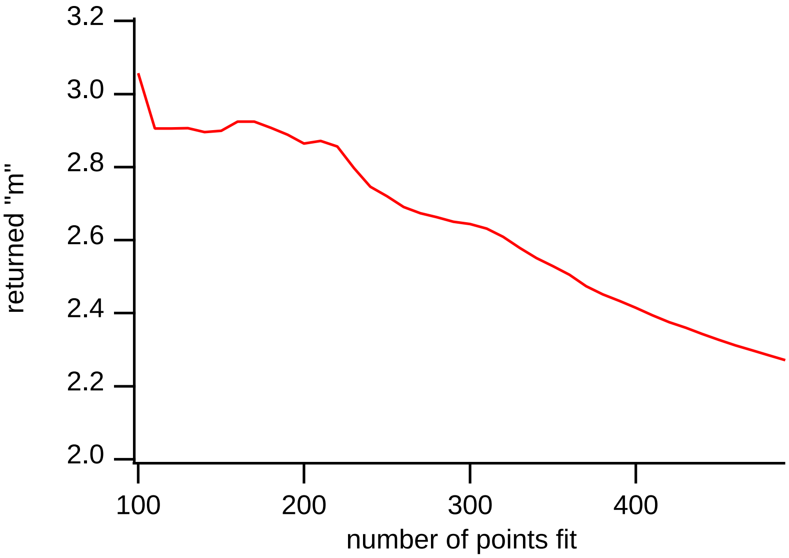

Figure S1.3
